# Primary, Secondary and Exploratory Endpoints in Phase 2 and 3 Clinical Trials with Novel Therapies in MASH Cirrhosis: A Systematic Review

**DOI:** 10.3390/jcm15072621

**Published:** 2026-03-30

**Authors:** Grzegorz Żurakowski, Anna Wiela-Hojeńska, Paweł Petryszyn

**Affiliations:** Department of Clinical Pharmacology, Wroclaw Medical University, 50-556 Wroclaw, Poland

**Keywords:** MASH, MASLD, non-invasive fibrosis scores, nonalcoholic steatohepatitis, liver cirrhosis, liver fibrosis, clinical trial endpoints, pharmacotherapy

## Abstract

**Background/Objectives:** Metabolic dysfunction-associated steatohepatitis (MASH) is a progressive liver disease and a major driver of cirrhosis, liver failure, and mortality worldwide. Despite the urgent need for effective therapies, clinical trials in MASH cirrhosis face substantial challenges due to the heterogeneity and the lack of standardization in study endpoints. A clear understanding of how endpoints are defined and applied across trials is critical for interpreting efficacy, comparing results, and guiding regulatory decisions. The objective of this systematic review was to classify and critically evaluate the primary, secondary, and exploratory endpoints used in phase 2 and 3 clinical trials of novel therapies for MASH cirrhosis, and to assess their consistency, strengths, and limitations. **Methods:** PubMed, Embase, and Cochrane Library databases were searched to identify Phase 2 and 3 trials of novel therapies for MASH-related cirrhosis. Studies of adults with biopsy-confirmed MASH cirrhosis in which clinical, histological, hemodynamic, imaging, and laboratory outcomes being assessed were included. **Results:** Nine eligible trials were included. Histological measures, most commonly improvement in fibrosis stage without worsening of MASH, were generally considered key efficacy outcomes. Biochemical (e.g., ALT, AST, Pro-C3, composite fibrosis scores) and imaging-based markers (e.g., liver stiffness) were widely used as secondary or exploratory endpoints and more frequently demonstrated treatment-related changes than histology. Hepatic venous pressure gradient (HVPG) was selected as a primary endpoint in some studies and as an exploratory outcome in others. Patient-centered outcomes, when incorporated, were typically exploratory. **Conclusions:** Phase 2 and 3 trials in MASH cirrhosis employ diverse and inconsistently defined endpoints, with limited standardization across studies. Establishing consensus on endpoint classification, definitions and clinical relevance is critical to advancing therapeutic development and ensuring regulatory acceptance in this high-risk patient population.

## 1. Introduction

Metabolic dysfunction-associated fatty liver disease (MASLD) and its severe form, metabolic dysfunction-associated steatohepatitis (MASH), represent a significant global health burden. MASLD is characterized by excessive intrahepatic fat accumulation unrelated to alcohol consumption [1]. MASH, the progressive form of MASLD, is characterized by inflammation and hepatocyte injury in addition to steatosis [2]. Cirrhosis represents the terminal stage of various chronic liver diseases, where progressive fibrosis leads to portal hypertension, hepatic decompensation, and finally hepatocellular carcinoma, resulting in increased morbidity and mortality [3,4].

The global prevalence of MASLD is approximately 35%, with MASH affecting 3–5% of the worldwide population [5]. Risk factors such as obesity, type 2 diabetes, and metabolic syndrome are closely associated with the development and progression of MAFLD and MASH [6]. The rising global obesity epidemic is expected to further increase MASLD and MASH incidence in coming years [7].

The pathophysiology of MASH involves multiple interrelated mechanisms contributing to hepatocyte injury, inflammation, and fibrosis. The multiple-hit hypothesis describes MASH progression in sequential stages. Initially, hepatic triglyceride accumulation causes steatosis and increases hepatocyte susceptibility to injury. Subsequent mechanisms include oxidative stress, characterized by excessive reactive oxygen species (ROS) production that induces hepatocyte damage, lipotoxicity from free fatty acid and metabolite accumulation, promoting cellular injury and apoptosis; and mitochondrial dysfunction, which impairs energy metabolism, elevates ROS levels, and contributes to cell death. Chronic inflammation results from activation of resident immune cells, such as Kupffer cells, and recruitment of additional immune cells that secrete pro-inflammatory cytokines and chemokines. Dysfunction of adipose tissue also plays a key role in this process: in the setting of insulin resistance and metabolic syndrome, hypertrophic adipocytes and infiltrating immune cells promote a systemic pro-inflammatory state and alter adipokine signaling, characterized by reduced adiponectin and increased leptin levels. In addition, gut dysbiosis, defined as alterations in gut microbiota composition, increases intestinal permeability, allowing endotoxins to translocate into the portal circulation and trigger hepatic inflammation. Collectively, these processes drive progressive liver injury, persistent inflammation, and the activation of hepatic stellate cells, which are crucial to the development of fibrosis [8].

Despite its high prevalence and potential to progress to severe liver disease, current management strategies for MASH primarily focus on lifestyle modifications—such as weight loss through diet and exercise—and on managing associated comorbidities [9]. However, these measures often prove insufficient for achieving long-term disease control, underscoring the unmet need for effective pharmacological treatments for MASH.

Insulin resistance is prevalent in both type 2 diabetes and obesity, serving as a key pathogenic driver to MASH progression. Due to obesity and the presence of metabolic and cardiovascular comorbidities, many patients with MASH are not eligible for liver transplantation, which remains the only curative option for progressive MASH-related cirrhosis [9,10].

Recent regulatory advances have marked an important step in the pharmacological management of metabolic dysfunction-associated steatohepatitis (MASH). In March 2024, the U.S. Food and Drug Administration (FDA) granted accelerated approval to resmetirom (Rezdiffra^®^ Madrigal Pharmaceuticals, Conshohocken, PA, USA), a selective thyroid hormone receptor-β agonist, for adults with non-cirrhotic MASH and moderate-to-advanced fibrosis (stages F2–F3) [11]. More recently, in August 2025, the FDA approved semaglutide (Wegovy^®^ Novo Nordisk, Bagsværd, Denmark), a glucagon-like peptide-1 receptor agonist previously used for obesity and cardiometabolic risk reduction, for the treatment of adults with MASH and moderate-to-advanced fibrosis [12]. Despite these advances, no pharmacological therapy has yet been approved specifically for patients with MASH-related cirrhosis (stage F4), and treatment options in this population remain limited.

Growing understanding of MASH pathophysiology has led to the identification of numerous potential therapeutic targets. As a result, a wide range of investigational therapies are currently being evaluated in preclinical and clinical studies. These agents target distinct biological pathways involved in MASH progression, which has led to the use of diverse efficacy endpoints in clinical trials.

Metabolic modulators (e.g., glucagon-like peptide-1 (GLP-1) receptor agonists, thyroid hormone receptor β agonists, fibroblast growth factor 21 (FGF21) analogues) primarily target insulin resistance, lipid metabolism, and weight reduction. Trials of these agents frequently assess steatohepatitis resolution, serum aminotransferase levels, and metabolic parameters as primary or secondary endpoints [13,14,15,16].

Antifibrotic therapies (e.g., apoptosis signal–regulating kinase 1(ASK1) inhibitors, lysyl oxidase-like 2 (LOXL2) inhibitors, galectin-3 antagonists) aim to directly reduce collagen deposition and fibrogenesis, with outcomes commonly including histological fibrosis stage, hepatic venous pressure gradient (HVPG), and composite fibrosis scores [17,18,19,20,21,22,23,24].

Bile acid pathway modulators (e.g., farnesoid X receptor (FXR) agonists like obeticholic acid or cilofexor) act by regulating bile acid synthesis and signaling. Endpoints typically include improvements in fibrosis, biochemical markers, and non-invasive imaging measures [25,26,27].

Apoptosis and inflammation inhibitors (e.g., pan-caspase inhibitors) target hepatocyte injury and inflammatory pathways, with endpoints reported as MELD score, Child–Pugh class, and biochemical indices of liver function [28,29,30,31].

This heterogeneity of mechanisms and associated outcomes underscores the central challenge in MASH cirrhosis trials: endpoints are reported in numerous ways, which complicates cross-trial comparison and regulatory interpretation. Regulatory agencies currently approve histological surrogates—specifically, ≥1-stage fibrosis improvement without worsening of steatohepatitis, or resolution of steatohepatitis without progression of fibrosis—as acceptable primary outcomes for conditional approval [11]. Intermediate markers, such as reductions in liver enzyme activities or improvements in hepatic fat content assessed by imaging, may also be considered if supported by substantial evidence linking them to long-term clinical outcomes. More recently, hepatic venous pressure gradient (HVPG) has gained recognition as a clinically meaningful endpoint, given its prognostic value for portal hypertension-related complications, including variceal bleeding. The Baveno VII consensus further supports HVPG as a validated outcome measure in advanced liver disease, offering a direct and reproducible assessment of treatment effects on hepatic hemodynamics [32].

However, despite a rapidly expanding therapeutic pipeline and the recognition of multiple surrogate and clinical outcomes, there is no standardized method for endpoint classification in MASH cirrhosis trials. The inconsistent labeling of outcomes as primary, secondary, or exploratory complicates comparisons across studies and makes interpretation of efficacy signals more challenging. The aim of the present study was to systematically identify, categorize, and critically assess the endpoints reported in phase 2 and 3 clinical trials of new therapies for MASH cirrhosis, with particular focus on their consistency across studies.

## 2. Materials and Methods

This systematic review was conducted and reported in accordance with the Preferred Reporting Items for Systematic Reviews and Meta-Analyses [33]. The PRISMA 2020 checklist is provided as Table S1. The review protocol was not pre-registered. A systematic search of the PubMed, Embase, and Cochrane Library databases was conducted to identify relevant studies published from 2018 to 4 April 2023. The starting year was chosen to focus on trials conducted under contemporary regulatory and methodological frameworks for MASH clinical research. The end date corresponds to the final date on which the database search was performed. The search strategy included the following terms: (‘nonalcoholic fatty liver’ OR ‘nonalcoholic steatohepatitis’ OR ‘metabolic dysfunction–associated fatty liver disease’ OR ‘metabolic dysfunction–associated steatotic liver disease’ OR ‘metabolic dysfunction–associated steatohepatitis’ OR ‘NAFLD’ OR ‘NASH’ OR ‘MAFLD’ OR ‘MASLD’ OR ‘MASH’) AND (‘liver cirrhosis’ OR ‘cirrhosis’:) AND (‘randomized controlled trial’ OR ‘controlled clinical trial’). The inclusion criteria were as follows: (1) phase II or III randomised clinical trials, (2) population were defined as adults with biopsy-confirmed NASH/MASH-related cirrhosis, (3) therapeutic intervention included drug therapies and (4) at least one of the following endpoints should have been assessed: liver fibrosis (e.g., ELF score, liver stiffness), markers of liver injury (e.g., ALT, AST), cardiometabolic risk parameters (weight loss, glycaemic control, and lipids), HVPG or decompensation events. The exclusion criteria included the following: (1) animal research, (2) commentaries, conference abstracts, editorials, case reports, (3) non-randomized controlled trial, (4) non-drug interventions. We decided to include only studies enrolling patients with established cirrhosis, as endpoint selection and therapeutic evaluation in advanced-stage disease remain particularly heterogeneous and less standardized compared with earlier stages of MASH. At this stage, the assessment of treatment efficacy increasingly depends on clinically meaningful liver-related outcomes. If duplicate research articles on the same cohort from the same period were identified, only the articles with the most comprehensive data available were included.

The process of data extraction was independently executed by two investigators (GZ and PP). The dataset comprised specific details about NASH/MASH cirrhosis, attributes of the sample group, and the main findings in terms of endpoints assessed and their performance in each individual study. In the event of discrepancies that emerged during the stages of data extraction and quality appraisal, a resolution was sought via a consensus-based approach, involving dialogue between the two investigators (GZ and PP) and an additional researcher (AWH). Specifically, when a disagreement occurred between the two primary researchers (GZ and PP), the third investigator (AWH) independently collected the relevant data, carried out the quality appraisal, and guided the subsequent discussion aimed at achieving consensus. The evaluation of the quality of enrolled RCTs was executed using the Cochrane risk of bias tool. This assessment encompassed seven facets: generation of a random sequence, concealment of allocation, blinding of both participants and staff, blinding during outcome assessment, data pertaining to incomplete outcomes, reporting selectivity, and other potential biases. Each criterion was classified under “low risk,” “high risk,” or “unclear risk” of bias. The assessment was independently carried out by two authors (GZ and PP) [34,35].

The authors declare that generative artificial intelligence tools (ChatGPT, OpenAI) were used to assist with language editing, stylistic refinement, and formatting of the manuscript. Different versions of ChatGPT were used during the manuscript preparation process, with the final editing performed using ChatGPT (version GPT-5.3, OpenAI, San Francisco, CA, USA). These tools were not used to generate scientific content, perform data analysis, interpret results, or draw conclusions. All content was critically reviewed and approved by the authors, who take full responsibility for the manuscript.

## 3. Results

### 3.1. Study Selection

**Figure 1:**
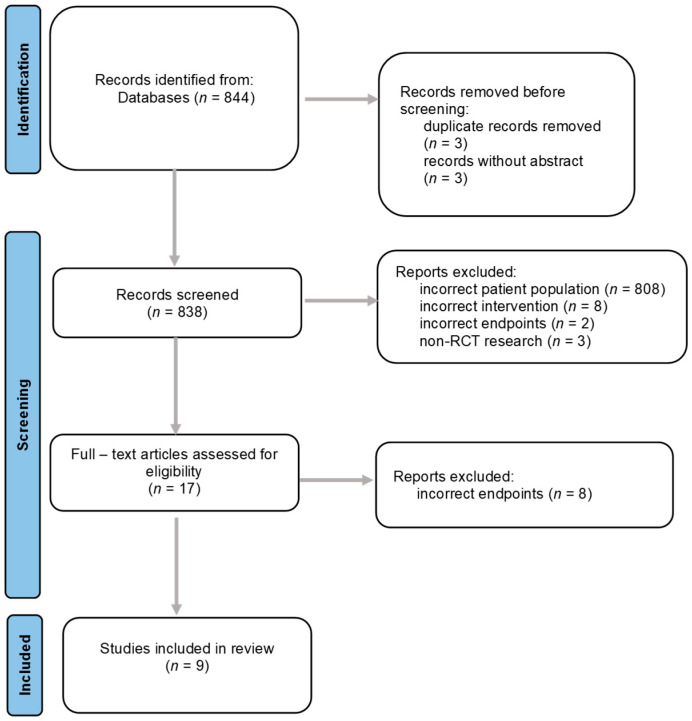
Flowchart of the literature screening process.

Figure 1 shows the details of the selection process: the initial search strategy yielded 844 records. After removing duplicates and records without abstracts, 838 records remained. These were screened based on their titles and abstracts, leading to the exclusion of 821 records that did not meet the criteria. A full-text review of the remaining 17 articles was conducted, eight articles were excluded at this stage because they did not include histological, biochemical, imaging, hemodynamic, or clinical endpoints related to treatment efficacy in MASH cirrhosis. Ultimately, nine articles were included in the review.

### 3.2. Study Characteristics and Quality Assessment

Table 1 summarizes the general characteristics of the nine included randomized controlled trials. Eight studies were phase II RCTs [23,24,30,36,37,38,39,40], and one was a phase III RCT [41]. The interventions represented a range of pharmacological mechanisms targeting different pathways involved in MASH progression. These included the pan-caspase inhibitor emricasan (three articles) [30,38,40], the glucagon-like peptide-1 receptor agonist semaglutide [36], the fibroblast growth factor-21 analogue efruxifermin [37], the galectin-3 antagonist belapectin [23], the lysyl oxidase-like 2 inhibitor simtuzumab [24], the apoptosis signal–regulating kinase 1 inhibitor selonsertib [39,41], the farnesoid X receptor agonist cilofexor [39], and the acetyl-CoA carboxylase inhibitor firsocostat [39] (one article each). In all studies, the control group received a placebo. All patients had clinically diagnosed MASH-related cirrhosis. Study follow-up ranged from 16 weeks [32] to 96 [26] weeks.

**Figure 2:**
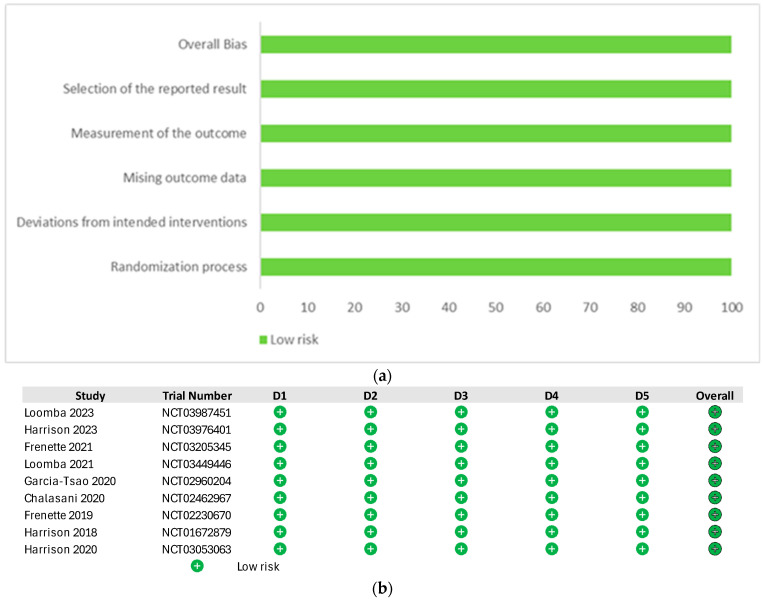
(**a**) Risk of bias graph and (**b**) Risk of bias summary. D1 Randomisation process, D2 Deviations from the intended interventions D3, Missing outcome data D4, Measurement of the outcome, D5 Selection of the reported result [23,24,30,36,37,38,39,40,41].

The quality of included studies, assessed using the Cochrane risk-of-bias tool, was generally high, with most trials reporting adequate randomization and blinding procedures (RoB), as detailed in Figure 2 [23,24,30,36,37,38,39,40,41].

### 3.3. Primary Endpoints

Primary endpoints differed across the included trials and largely reflected the intended mechanism of action of the investigated therapies. In studies focusing on antifibrotic strategies in MASH cirrhosis, histological outcomes assessed on liver biopsy were most commonly selected (Table 1). The most frequently used endpoint was improvement in liver fibrosis by at least one stage (commonly interpreted as histological regression of cirrhosis) without worsening of steatohepatitis, evaluated using standardized systems such as NASH CRN or Ishak scoring. This endpoint served as the primary efficacy measure in trials of selonsertib [41] and simtuzumab [24]. In the phase 3 selonsertib trial [41], the predefined histological endpoint was not met after 48 weeks, with similar proportions of responders in the active treatment and placebo groups. Likewise, in the simtuzumab study [24], repeat biopsy at week 96 did not demonstrate a meaningful difference in fibrosis stage or collagen content compared with placebo, despite long treatment duration and parallel hemodynamic assessment.

Not all trials selected histology as the primary endpoint. In studies primarily addressing portal hypertension and the risk of clinical decompensation, hemodynamic assessment of portal pressure was prioritized instead. In particular, hepatic venous pressure gradient (HVPG) was used as the primary endpoint in trials evaluating emricasan [40] and belapectin [23], reflecting its established prognostic relevance in advanced cirrhosis. In the emricasan trial [40] enrolling patients with clinically significant portal hypertension, the primary endpoint was the change in HVPG from baseline to week 24. This endpoint was not met, as no significant reduction in portal pressure was observed compared with placebo. Similarly, in the belapectin study [23], mean change in HVPG after 52 weeks of treatment did not differ significantly between treatment and placebo groups. Although prespecified subgroup analyses suggested a reduction in HVPG and fewer new varices among patients without baseline varices, these findings did not translate into a positive primary outcome for the overall population.

Several trials adopted composite clinical endpoints as primary measures, particularly in populations with more advanced or decompensated disease. In the phase 2 emricasan study by Frenette et al. [30], the primary endpoint was time to first clinical event, defined as a composite of all-cause mortality, new or worsening hepatic decompensation, or MELD-Na progression. This endpoint was not met, with similar event rates observed across treatment and placebo arms. MELD-Na progression was the most common first event, and emricasan did not demonstrate a protective effect on liver-related outcomes.

In contrast to efficacy-driven primary endpoints, some early phase studies designated safety and tolerability as their primary objective. In the efruxifermin phase 2 trial [37], the primary endpoint was safety and tolerability in patients with compensated MASH cirrhosis. While the study met this primary endpoint and demonstrated a favorable safety profile, efficacy signals were evaluated primarily through secondary and exploratory endpoints, including non-invasive markers of fibrosis and metabolic parameters.

Across the nine included randomized controlled trials, only a minority met their predefined primary efficacy endpoints. Specifically, two studies achieved their primary endpoint, whereas seven trials did not demonstrate statistically significant improvement compared with placebo within the predefined follow-up periods. In several trials, however, statistically significant changes were observed in secondary or exploratory outcomes, particularly in metabolic parameters, biochemical markers of liver injury, or non-invasive fibrosis markers. A detailed overview of primary, secondary, and exploratory endpoints across individual trials is presented in Table 1.

Taken together, primary endpoints across the included trials can be classified into three broad categories: histological cirrhosis regression, hemodynamic improvement assessed by HVPG, and composite clinical or safety-based outcomes. Although a small number of studies achieved their predefined primary endpoints, most trials did not demonstrate statistically significant improvement versus placebo within the predefined follow-up period. This pattern highlights the biological and methodological challenges of demonstrating treatment efficacy in MASH-related cirrhosis, particularly within the relatively short duration of phase 2 and early phase 3 trials.

### 3.4. Secondary Endpoints

Secondary endpoints differed across trials and included non-invasive imaging measures, biochemical markers of liver injury and fibrosis, and functional liver scores (Table 1).

Non-invasive imaging was reported as a secondary endpoint in a subset of studies. Liver stiffness assessed by vibration-controlled transient elastography or magnetic resonance-based techniques was evaluated in metabolic-targeting trials, including studies of efruxifermin and combination therapy with cilofexor and firsocostat [39]. In these trials, reductions in liver stiffness or hepatic fat content were reported in active treatment arms compared with placebo. By contrast, antifibrotic trials evaluating simtuzumab [24] or selonsertib [41] did not demonstrate significant differences in imaging-based measures between treatment and placebo groups, or included imaging only as exploratory outcomes.

Biochemical secondary endpoints were reported in all included trials. Serum alanine aminotransferase and aspartate aminotransferase were assessed consistently, with several studies reporting statistically significant reductions in their activities compared with placebo. Composite fibrosis markers, including the enhanced liver fibrosis score, Pro-C3, and TIMP-1, were evaluated in selected trials and showed variable responses across studies. These biochemical changes were not consistently accompanied by histological improvement in fibrosis. One possible explanation is that metabolic and inflammatory markers often respond relatively quickly to pharmacological intervention, whereas regression of advanced fibrosis in cirrhosis represents a slower structural process that may require longer observation periods to become detectable on liver biopsy. In addition, the inherent sampling variability of liver biopsy may further limit the ability to capture modest structural changes within the relatively short time frames of most clinical trials.

Functional liver scores were included as secondary endpoints in selected trials in MASH-related cirrhosis. The model for End-Stage Liver Disease and Child–Pugh scores were reported in studies evaluating emricasan [30,38,40], belapectin [23], and simtuzumab [24]. Across trials, mean changes in these scores were minimal, and no consistent differences versus placebo were observed. Taken together, secondary endpoints varied substantially across trials and were more likely to show treatment-related changes in biochemical and imaging measures than in functional liver scores.

### 3.5. Exploratory Endpoints

Exploratory endpoints were prespecified in only a subset of the included trials and varied substantially between studies (Table 2). These endpoints most often comprised non-invasive laboratory test and, less frequently, imaging-based biomarkers of steatosis, inflammation, and fibrosis. Across trials, these exploratory measures demonstrated limited or inconsistent changes and were not uniformly associated with clear treatment effects at the study level.

The most frequently assessed parameters included fibrosis markers (such as ELF score, Pro-C3, and TIMP-1), markers of hepatocellular injury or inflammation (including aminotransferases, hsCRP, and PAI-1), and imaging-derived measures of liver fat or stiffness. In some trials, statistically significant differences between treatment and placebo groups were observed for individual biomarkers. For example, efruxifermin [37] was associated with reductions in ELF score and Pro-C3, while combination therapy with cilofexor and firsocostat resulted in improvements in selected non-invasive fibrosis markers and imaging-based parameters [39]. However, these effects were not consistently replicated across studies and were not accompanied by concordant improvements in primary endpoints or clinical outcomes.

In the emricasan trials, apoptosis-related biomarkers were evaluated as secondary and exploratory endpoints. In the phase 2 studies by Frenette et al. (2019) [30] and Garcia-Tsao et al. (2020) [40], these included caspase-3/7 activity and cytokeratin-18 fragments (cleaved and full-length). Reductions in these markers were observed in some treatment arms, particularly at higher doses, and earlier time points; however, these changes were not linked to consistent improvements in clinical outcomes or liver histology.

In the belapectin trial [23], the primary endpoint was the mean change in hepatic venous pressure gradient (HVPG) from baseline compared with placebo. In contrast, exploratory analyses focused on HVPG response stratified by baseline portal hypertension severity and the presence of esophageal varices, applying categorical rather than continuous definition of response. Within these subgroup analyses, a numerical reduction in HVPG was observed among patients without baseline varices, whereas no significant effect was detected in the overall study population.

Additional exploratory endpoints included functional liver scores (Model for End-Stage Liver Disease and Child–Pugh classification), liver-related clinical events, and patient-reported outcomes (PROs). Functional scores and liver events generally showed minimal or no differences between treatment and placebo groups. Patient-reported outcomes were rarely explored among the exploratory endpoints. Where assessed, quality-of-life measures were not prespecified within the statistical hierarchy and were reported descriptively.

Taken together, exploratory endpoints provided supplementary mechanistic and subgroup-level information but were heterogeneously defined and inconsistently reported across trials.

## 4. Discussion

**Figure 3:**
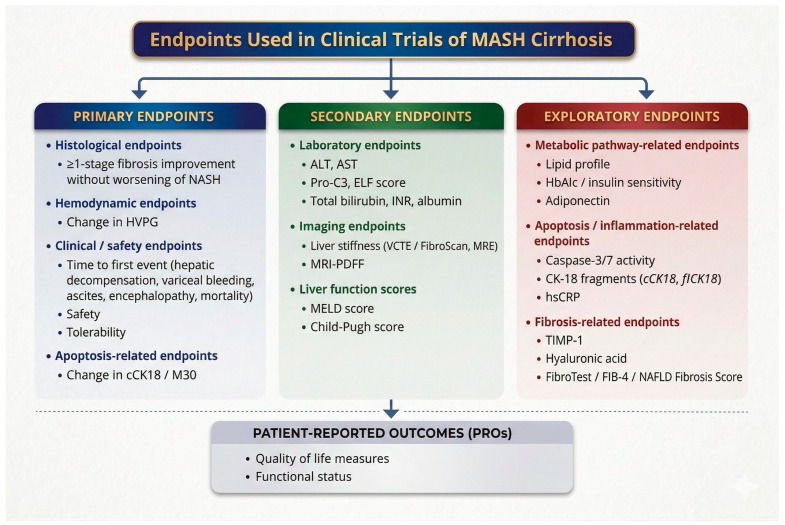
Conceptual classification of primary, secondary and exploratory endpoints used in clinical trials of therapies for MASH-related cirrhosis.

This systematic review provides a structured synthesis of primary, secondary, and exploratory endpoints reported in phase 2 and 3 randomized trials of novel therapies for MASH-related cirrhosis. Across the nine included studies, endpoints encompassed histological assessments of fibrosis, non-invasive biomarkers, hemodynamic parameters, clinical scores, and, more rarely, patient-reported outcomes. By explicitly classifying endpoints according to their hierarchical role and mapping them across trials, this review highlights both the breadth of outcome measures currently employed and the substantial lack of harmonization in endpoint selection and reporting in this advanced disease stage. Given the heterogeneity of endpoints reported across the included trials, a conceptual framework categorizing primary, secondary, and exploratory outcomes in clinical studies of therapies for MASH cirrhosis is presented in Figure 3.

A key contribution of this review is the demonstration that endpoint hierarchy is often poorly defined or inconsistently reported. While some trials clearly prespecified primary, secondary, and exploratory outcomes, others lacked explicit designation, requiring inference based on study objectives or statistical plans. This inconsistency complicates cross-trial comparisons and limits the interpretability of negative or discordant results. From a methodological standpoint, clearer and more transparent reporting of endpoint hierarchy is essential to strengthen the evidence base in MASH-related cirrhosis.

Histological outcomes, particularly ≥1-stage fibrosis improvement without worsening of steatohepatitis, remained the most commonly used primary endpoints, reflecting current regulatory paradigms. However, as demonstrated in multiple trials—including the phase III selonsertib program [41] and simtuzumab studies [24]—histology-based endpoints failed to demonstrate treatment benefit in MASH cirrhosis. These findings underscore the biological and technical limitations of liver biopsy in advanced disease, where fibrosis regression is slow, sampling variability is substantial, and short- to mid-term changes may not translate into meaningful clinical benefits. While histology remains an accepted surrogate, its utility as a sole primary endpoint in cirrhosis appears constrained.

Biochemical and imaging-based markers were widely used as secondary or exploratory endpoints and more frequently demonstrated treatment-related changes than histology. Markers such as ELF score, Pro-C3, aminotransferases, and imaging-derived liver stiffness or fat content often moved in a favorable direction, even when primary histological endpoints were not achieved. These observations suggest that non-invasive tests (NITs) may capture pharmacodynamic effects earlier and with greater sensitivity than biopsy in cirrhosis. However, heterogeneity in marker selection, cut-off definitions, and assessment timing across trials limits comparability and prevents firm conclusions regarding their validity as surrogate endpoints for clinical outcomes.

Despite their established prognostic value in cirrhosis, hemodynamic and clinical endpoints were used sparingly. HVPG was selected as a primary endpoint in few studies and as an exploratory outcome in others. The belapectin trial [23] illustrates both the potential and the limitations of this approach: while subgroup analyses suggested a numerical HVPG reduction in patients without baseline varices, the primary endpoint was not met in the overall population. Similarly, clinical scores (MELD, Child–Pugh) and decompensation events were inconsistently reported and seldom powered to detect meaningful differences. Given that these endpoints are most directly linked to patient prognosis, their limited and inconsistent inclusion represents a major gap in current trial design.

Exploratory endpoints provided valuable mechanistic and subgroup-level insights, frequently demonstrating target engagement; however, they did not consistently correlate with histological, hemodynamic, or clinical improvements.

Across all included trials, patient-reported outcomes (PROs) were notably underrepresented. When evaluated, quality-of-life measures were typically exploratory, descriptive, and not integrated into the statistical hierarchy. Given the substantial symptom burden and functional impairment experienced by patients with cirrhosis, the lack of robust patient-centered outcomes limits our understanding of how therapies affect daily life and overall well-being. Incorporating patient-reported outcomes (PROs) alongside biological and clinical endpoints would provide a more comprehensive evaluation of therapeutic value.

The strengths of this review include a systematic approach focused specifically on MASH-related cirrhosis, detailed endpoint classification, and cross-trial synthesis informed by original study reports. Limitations include the relatively small number of available trials, heterogeneity in study design and follow-up duration, and reliance on published data, which may incompletely capture endpoint hierarchies or negative results.

In conclusion, the evidence synthesized in this review indicates that although therapeutic development in MASH cirrhosis is progressing, endpoint selection remains fragmented and insufficiently aligned with disease biology and patient priorities. Our findings support the need for a more standardized and transparent endpoint framework, with clear distinction between primary, secondary, and exploratory outcomes. Future trials should consider composite strategies that integrate validated non-invasive biomarkers, hemodynamic assessment, and clinically meaningful outcomes such as decompensation events and transplant-free survival, along with patient-reported measures. Consensus-driven efforts—potentially through multi-society collaborations—will be essential to define core outcome sets suitable for cirrhosis-stage disease.

## Figures and Tables

**Table 1:** Summary of the included studies.

Trial (Year/NCT)	Agent (vs. Placebo)	Population & Duration	Primary Endpoint	Secondary Endpoints	Exploratory Endpoints	Key Findings vs. Placebo
Loomba, 2023 (NCT03987451) Phase 2 [36]	semaglutide 2.4 mg	adults with biopsy-confirmed NASH-related cirrhosis and body-mass index (BMI) of 27 kg/m^2^ or more; 48 weeks	improvement in liver fibrosis by ≥1 stage without worsening of NASH (NASH CRN)	MRI-PDFF, MRE, NASH resolution on biopsy, TEAEs	EFL, Pro-C3, FIB-4, TIMP-1, Total adiponectin, Hyaluronic acid, hsCRP, ALT, AST, GGTP, MELD, Child-Pugh, albumin, billirubin, INR, platelets	Primary endpoint not met; significant metabolic and biochemical improvements
Harrison, 2023 (NCT03976401) Phase 2 [37]	efruxifermin 50 mg	adults with NASH and compensated cirrhosis (F4 fibrosis); 16 weeks	safety and tolerability of efruxifermin in patients with compensated NASH cirrhosis	liver stiffness (FibroScan), Pro-C3, ELF	ALT, AST, GGTP, ALP, bilirubin, INR, urate, albumin, MELD, Child–Pugh, triglycerides, lipoprotein profile, HbA1c, insulin sensitivity, hsCRP, platelets, CAP-assessed steatosis, NAS components, fibrosis regression (NASH CRN), Pro-C3/C3M ratio	Primary endpoint met; significant reductions in liver fat and liver stiffness with associated biochemical improvements.
Frenette, 2021 (NCT03205345) Phase 2 [38]	emricasan 5 or 25 mg	adults with NASH cirrhosis and history of variceal hemorrhage or history of at least moderate ascites requiring current diuretic treatment; 48 weeks	time to first event (composite): all-cause mortality; new decompensation event (new or recurrent variceal hemorrhage, new ascites requiring diuretics, new unprecipitated hepatic encephalopathy ≥ grade 2 requiring hospitalization, hepatorenal syndrome requiring hospitalization, spontaneous bacterial peritonitis requiring hospitalization); or increase in MELD-Na score ≥ 4 points from baseline.	composite clinical endpoint excluding MELD-Na progression;	caspase 3/7, cCK18, flCK18, ALT, AST, MELD-Na, CTP, total bilirubin, INR, serum albumin	Primary endpoint not met; no reduction in clinical events or MELD-Na progression; transient reductions in apoptosis biomarkers without corresponding clinical or histological benefit.
Loomba, 2021 (NCT03449446) Phase 2 [39]	selonsertib 18 mg, cilofexor 30 mg, firsocostat 20 mg	adults with biopsy-confirmed NASH with either bridging fibrosis (F3) or compensated cirrhosis (F4); 48 weeks	≥1-stage improvement in fibrosis without worsening of NASH (NASH CRN)	Not formally specified	NASH resolution, NAS improvement, ALT, AST, bile acids, CK18 (M30/M65), ELF score, liver stiffness (VCTE), MRI-PDFF (subset)	Primary endpoint not met; combination therapy (cilofexor/firsocostat) associated with significant improvements in NASH activity, liver enzymes, noninvasive fibrosis markers, and liver stiffness, without statistically significant histologic fibrosis regression
Garcia-Tsao, 2020 (NCT02960204) Phase 2 [40]	emricasan 5 or 25 or 50 mg	NASH cirrhosis patients with screening HVPG ≥ 12 mmHg; 24 weeks	mean Change in Hepatic Venous Pressure Gradient (HVPG)	liver stiffness (FibroScan), ELF, ALT, AST, Caspase 3/7	Decompensation events, MELD, Child-Pugh	Primary endpoint not met; no significant HVPG reduction; no effect on decompensation, MELD, or Child–Pugh; treatment well tolerated.
Chalasani, 2020 (NCT02462967) Phase 2 [23]	belapectin 2 or 8 mg/kg	patients with NASH, cirrhosis, and portal hypertension (hepatic venous pressure gradient [HVPG] ≥ 6 mm Hg); 52 weeks	mean Change in Hepatic Venous Pressure Gradient (HVPG)	Collagen proportionate area; ≥1-stage fibrosis change (Ishak/NASH CRN), liver stiffness, cirrhosis complications composite (variceal bleeding, ascites/SBP, hepatic encephalopathy, Child–Pugh, variceal progression, MELD, transplant, liver-related mortality)	HVPG response in MPH vs. CSPH; TEAE, SAEs	Primary endpoint not met; no overall improvement in HVPG or fibrosis; HVPG reduction and fewer new varices in patients without baseline varices.
Frenette, 2019 (NCT02230670) Phase 2 [30]	emricasan 25 mg	adults with NASH cirrhosis; 26 weeks	change CCK-18/M30	total bilirubin, INR, albumin, MELD, Child-Pugh	ALT, caspase 3/7, CK-18, flCK-18, cCK-18	Primary endpoint met in patients with higher baseline MELD; no histological improvement; caspase activity reduced without consistent clinical benefit.
Harrison, 2018 (NCT01672879) Phase 2 [24]	simtuzumab 200 or 700 mg	patients with compensated cirrhosis; 96 weeks	Change in HVPG	Not formally specified	Hepatic collagen content, Fibrosis stage (Ishak; NASH CRN), liver-related clinical events, ALT, AST, GGT, ALP, bilirubin, INR, albumin, LOXL2, ELF, FibroTest, MELD, NAFLD Fibrosis Score, Event-free survival	Primary endpoint not met; no significant reduction in HVPG or fibrosis; no improvement in clinical outcomes.
Harrison, 2020 (NCT03053063) Phase 3 [41]	selonsertib 6 or 18 mg	adults with biopsy-confirmed NASH with compensated cirrhosis (F4); 48 weeks	≥1-stage improvement in fibrosis without worsening of NASH	proportions of patients with a ≥1-stage improvement in fibrosis, the proportion of patients with NASH resolution (lobular inflammation score of 0–1, ballooning 0)	Fibrosis stage (Ishak; NASH CRN), hepatic collagen, ALT, AST, GGT, ALP, total bilirubin, INR, albumin, bile acids, ELF, FibroTest, FIP-4, NAFLD Fibrosis Score, CK18 (M30/M65), MELD, Child-Pugh, Cholesterol total, LDL, HDL, triglycerides, glucose, insulin, HbA1c, liver stiffness (VCTE)	Primary endpoint not met; no antifibrotic effect; no significant improvement in liver biochemistry, NITs, or adjudicated clinical events.

**Table 2:** Performance of biochemical, imaging, and clinical endpoints across included trials.

Endpoint/Study	NCT03987451	NCT03976401	NCT03205345	NCT03449446	NCT02960204	NCT02462967	NCT02230670	NCT01672879	NCT03053063
	Loomba, 2023 [36]	Harrison, 2023 [37]	Frenette, 2021 [38]	Loomba, 2021 [39]	Garcia-Tsao, 2020 [40]	Chalasani, 2020 [23]	Frenette, 2019 [30]	Harrison, 2018 [24]	Harrison, 2020 [41]
ALT, U/L	x ↓ SEM vs. placebo * 0.76 (0.61–0.93) **	x ↓ EFX vs. placebo * −10.3 (−14.3, −6.4) ***	x	x ↓ CILO/FIR vs. placebo * −18 (−24, −12) ***	x ↓ EMR5 vs. placebo * 0.8346 (0.7533, 0.9247) *** EMR25 vs. placebo * 0.8279 (0.7472, 0.9173) *** EMR50 vs. placebo * 0.8658 (0.7809, 0.96) ***		x	x	x
AST, U/L	x ↓ SEM vs. placebo * 0.77 (0.65–0.92) **	x	X ↓ EMR25 vs. placebo *	x ↓ CILO/FIR vs. placebo * −12 (−17, −7) ***	x ↓ EMR5 vs. placebo * 0.892 (0.8141, 0.9772) *** EMR25 vs. placebo * 0.8683 (0.7926, 0.9513) ***		x	x	x
GGTP, U/L	x ↓ SEM vs. placebo * 0.74 (0.62–0.88) **	x		x		32		x	x
ALP, U/L		x		x ↓ CILO/FIR vs. placebo * 19 (10, 29) ***				x	x
Urate, mg/dL		x ↓ EFX vs. placebo * −0.98 (−1.32, −0.63) ***							
Bilirubin, mg/dL	x	x	x	x ↓ CILO vs. placebo * 0.0 (−0.1, 0.0) *** FIR/SEL vs. placebo * −0.1 (−0.1, 0.0) *** CILO/FIR vs. placebo * −0.1 (−0.1, 0.0) ***	x ↓ EMR all vs. placebo * −3.6 (19.1) vs. +0.3 (14.3)		x	x	x
Fibrinogen, mg/dL		x ↓ EFX vs. placebo * 29.3 ***							
INR	x	x	x		x		x EMR vs. placebo * −0.13 (−0.6, 0.35) ***		x
MELD	x	x	x		x		x EMR vs. placebo * −1.6 (−2.6, −0.6) ***	x	x
Child-Pugh score	x	x	x		x		x EMR vs. placebo * −1.0 (−1.7, −0.3) ***		
eGFR, mL/min/m^2^	x			x ↓ CILO/FIR vs. placebo * 4.5 (1.6, 7.4) ***					
HDL-C, mg/dL	x	x ↑ EFX vs. placebo * 33%							x
LDL-C, mg/dL	x	x ↓ EFX vs. placebo * −8%							x
Triglycerides, mg/dL	x ↓ SEM vs. placebo * 0.834 (0.723–0.962) **	x ↓ EFX vs. placebo * −29%							x
VLDL mg/dL	x ↓ SEM vs. placebo * 0.833 (0.722–0.961) **								
Non-HDL-C, mg/dL		x ↓ EFX vs. placebo * −14%							
Apolipoprotein B, mg/dL		x ↓ EFX vs. placebo * −11.1%							
Lipoprotein-a (nmol/L)		x ↑ EFX vs. placebo * 32.7%							
Liver steatosis, MRI-PDFF, %	x ↓ SEM vs. placebo * 0.67 (0.51–0.88) **			x ↓ FIR vs. placebo * −0.1 (−0.4, 0.1) *** CILO/FIR vs. placebo * −0.0 (−0.2, 0.2) ***					
Liver fat volume, L	x ↓ SEM vs. placebo * 0.58 (0.42–0.81) **								
Hyaluronic acid, µg/L	x	x ↓ EFX vs. placebo * −19.4 (−58.3, 6.4) ***							
P3NP, µg/L	x	x ↓ EFX vs. placebo * −3.2 (−5.7, −1.2) ***							
Pro- C3, µg/L	x ↓ SEM vs. placebo * 0.844 (0.727–0.980) **	x ↓ EFX vs. placebo * −9.0 (−11.5, −6.5) ***							
TIMP1, µg/L	x ↓ SEM vs. placebo * 0.891 (0.798–0.995) **	x ↓ EFX vs. placebo * −35.7 (−74.1, 3.6) ***							
ELF score		x ↓ EFX vs. placebo * −0.4 (−0.9, −0.0) ***		x ↓ FIR vs. placebo * −2.96 (−5.67, −0.24) *** FIR/SEL vs. placebo * −3.70 (−5.71, −1.69) *** CLIO/SEL vs. placebo * −2.03 (−3.83, −0.23) *** CILO/FIR vs. placebo * −4.00 (−6.01, −1.98) ***					x
Liver stiffness, MRE, kPa	x	x		x	x ↓ EMR all vs. placebo * −3.6 (19.1) vs. −0.3 (14.3)				
Total liver volume, L	x ↓ SEM vs. placebo * 0.87 (0.82–0.93) **								
hsCRP	x ↓ SEM vs. placebo * 0.592 (0.404–0.868) **	x							x
CASP3/7			x ↓ EMR25 vs. placebo *				x		
PAI-1, IU/mL		x ↓ EFX vs. placebo * −8.74 (1.7) ***							
cCK18, U/L			x	x ↓ FIR/SEL vs. placebo * −141 (−211, −72) *** CILO/FIR vs. placebo * −158 (−226, −90) ***	x ↓ EMR5 vs. placebo * 0.8348 (0.7409, 0.9406) *** EMR25 vs. placebo * 0.7752 (0.6881, 0.8733) *** EMR50 vs. placebo * 0.7687 (0.6821, 0.8662) ***		x		x
flCK18, U/L			x		x ↓ EMR5 vs. placebo * 0.8474 (0.7593, 0.9456) *** EMR25 vs. placebo * 0.7995 (0.7167, 0.8919) ***	x	x		x
Bile acids (lmol/L)		x		x ↓ CILO/FIR vs. placebo * −2.7 (−4.6, −0.8) ***					

x—biomarker was assessed in the study but did not reach statistical significance; blank cell—biomarker not assessed in the study; *—statistically significant difference vs. placebo; **—ETR—Estimated Treatment Ratio; ***—LS mean (95%CI).

## Data Availability

No new data were created or analyzed in this study. Data sharing is not applicable to this article.

## Supplementary Materials

The following supporting information can be downloaded at: https://www.mdpi.com/article/10.3390/jcm15072621/s1, Table S1: PRISMA 2020 Checklist.

## Abbreviations

The following abbreviations are used in this manuscript:

ALT	alanine aminotransferase
ASK1	apoptosis signal-regulating kinase 1
AST	aspartate aminotransferase
CASP3/7	caspase 3/7
CI	confidence interval
CILO	Cilofexor
cCK18	cleaved cytokeratin-18
ELF score	Enhanced Liver Fibrosis score
EFX	Efruxifermin
EMR	Emricasan
ETR	estimated treatment ratio
FGF21	fibroblast growth factor 21
FIR	Firsocostat
flCK18	full-length cytokeratin-18
FXR	farnesoid X receptor
GGTP	gamma-glutamyl transpeptidase
GLP-1	glucagon-like peptide-1
hsCRP	high-sensitivity C-reactive protein
HVPG	hepatic venous pressure gradient
LOXL2	lysyl oxidase-like 2
LS mean	least squares mean
MAFLD	metabolic dysfunction-associated fatty liver disease
MELD-Na	Model for End-Stage Liver Disease–sodium
MRI-PDFF	magnetic resonance imaging–proton density fat fraction
MRE	magnetic resonance elastography
NASH CRN	Nonalcoholic Steatohepatitis Clinical Research Network
PAI-1	plasminogen activator inhibitor-1
P3NP	procollagen type III N-terminal peptide
Pro-C3	N-terminal pro-peptide of type III collagen
PROs	patient-reported outcomes
RCTs	randomized controlled trials
RoB	risk of bias
ROS	reactive oxygen species
SEL	selonsertib
SEM	semaglutide
TIMP-1	tissue inhibitor of metalloproteinases-1
